# Mitochondrial Dysfunction in Atrial Fibrillation—Mechanisms and Pharmacological Interventions

**DOI:** 10.3390/jcm10112385

**Published:** 2021-05-28

**Authors:** Paweł Muszyński, Tomasz A. Bonda

**Affiliations:** Department of General and Experimental Pathology, Medical University of Bialystok, Mickiewicza 2c, 15-222 Bialystok, Poland; Pawel.Muszynski@umb.edu.pl

**Keywords:** atrial fibrillation, mitochondria, cardiac remodeling, pharmacotherapy

## Abstract

Despite the enormous progress in the treatment of atrial fibrillation, mainly with the use of invasive techniques, many questions remain unanswered regarding the pathomechanism of the arrhythmia and its prevention methods. The development of atrial fibrillation requires functional changes in the myocardium that result from disturbed ionic fluxes and altered electrophysiology of the cardiomyocyte. Electrical instability and electrical remodeling underlying the arrhythmia may result from a cellular energy deficit and oxidative stress, which are caused by mitochondrial dysfunction. The significance of mitochondrial dysfunction in the pathogenesis of atrial fibrillation remains not fully elucidated; however, it is emphasized by the reduction of atrial fibrillation burden after therapeutic interventions improving the mitochondrial welfare. This review summarizes the mechanisms of mitochondrial dysfunction related to atrial fibrillation and current pharmacological treatment options targeting mitochondria to prevent or improve the outcome of atrial fibrillation.

## 1. Introduction

Atrial fibrillation (AF) is the most frequent type of arrhythmia occurring, especially among patients with more advanced age. It is estimated that the prevalence of AF in the global population varies between 2% and 3.4% [1,2,3]. AF is 1.2 times more prevalent among males than females, less frequent at a younger age (0.12–0.16% > 49 y) and increases with age (3.7–4.2% 60–70 y and in 10–17% > 80 y.) [1]. The early onset of AF may be attributed to a genetic component or congenital heart defects [4,5,6]. In younger patients without structural heart defects, the pulmonary vein arrhythmogenesis frequently acts as the initiating trigger for AF, mostly paroxysmal, and recurrences can be successfully prevented by pulmonary vein ablation [7]. However, in elderly patients, the key initiating factors are atrial tissue degeneration and comorbidities affecting the atrial metabolism and atrial structure, more frequently leading to persistent or permanent AF [8,9].

AF significantly affects the quality of life, worsens morbidity and mortality and generates a significant socioeconomic burden [10]. Due to comorbidities caused by AF complications, a large proportion of people with AF are not able to function normally in society. Financial outlays for hospitalizations due to AF in 2005 cost USD 6.65 billion in the US [10]. The cost per patient per year was estimated from USD 2000 to USD 14200 in the US and from EUR 450 to EUR 3000 in Europe and was further increased by the introduction of non-vitamin K antagonist oral anticoagulants [11,12]. The aging of the population in developed countries will increase the number of people with AF. Therefore, advances in understanding the mechanisms leading to this arrhythmia for improving prevention and treatment are crucial to restrain the AF epidemic.

The pathophysiology of AF has been thoroughly investigated in the past decades, and the association between mitochondrial dysfunction and AF was proposed as early as the 1970s [13]. Mitochondria are organelles present in metabolically active cells, such as cardiomyocytes, in large numbers. They are responsible for synthesis of adenosine 5′-triphosphate (ATP), which provides energy for almost all intracellular processes, including mechanical work and active ion transport. Disturbance of mitochondrial energetics, oxidative stress and electrical remodeling was proposed to be associated with the occurrence of the arrhythmia [13,14]. Recent progress in scientific tools, including genomic, proteomic and metabolomic analyses, allow for further detailed investigation of the mechanisms linking consequences of mitochondrial dysfunction with development of AF [15,16,17,18].

This review summarizes the knowledge about the role of mitochondrial dysfunction in the pathogenesis of AF and gives an overview of the current treatment options to ameliorate the condition of mitochondria to prevent the arrhythmia or improve prognosis.

## 2. General Mechanisms of AF

The main mechanism of AF perpetuation is the re-entry phenomenon. It usually depends on the preexisting morphological substrate of enlarged atria, which are able to accommodate one or multiple depolarization waves or the spiral wave [19]. Premature atrial beats, originating most frequently from the myocardial sleeves within the pulmonary veins, trigger the re-entry and initiate AF [20]. However, in younger patients without significant morphological heart disease, rapid focal activity from the pulmonary veins may be more important as the underlying mechanism of AF. The arrhythmogenic foci may depend on diastolic calcium leak from the sarcoplasmic reticulum (SR) due to ryanodine receptor (RyR) hyperphosphorylation that promotes delayed afterdepolarizations and triggered activity to induce AF [21]. Re-entry and rapid focal activity both induce frequent atrial depolarizations and electrical remodeling that is characterized by slowed conduction and shortened atrial refractoriness, which promote re-entry and persistence of AF [20]. Electrical remodeling is induced by SR calcium overload and elevated cytoplasmic Ca^2+^levels, reduced expression of the slow inward calcium channels, increased rectifier potassium current and altered expression of the connexins [22].

Many clinical risk factors promoting the development of AF were identified, such as older age, male sex, obesity, hypertension, coronary artery disease, heart failure (HF), chronic kidney disease, hyperthyroidism, diabetes mellitus (DM), chronic obstructive pulmonary disease (COPD) and valvular heart disease [23,24,25]. These disorders are responsible for the induction of unfavorable atrial structural remodeling and formation of the morphological substrate sustaining the arrhythmia. The pathomechanisms of structural remodeling include atrial pressure and volume overload leading to atrial dilation (valvular heart disease, HF), increased atrial epicardial fat tissue (obesity, metabolic syndrome), autonomic nervous system dysfunction (diabetes mellitus), systemic inflammation (coronary artery disease, HF, COPD), increased fibrosis, cellular ultrastructural defects and contractile proteins dysfunction [24,26].

## 3. Role of Mitochondria in the Physiology of the Heart

The mechanical work of the cardiac muscle relies on the contractile apparatus built up by the filaments of actin, myosin and dozens of regulatory proteins packed together into sarcomeres. Cardiac myocyte’s contraction–relaxation cycle is coupled to the cytoplasmic calcium cycling, and both mechanical work and regulation of cytoplasmic calcium levels require uncompromised delivery of ATP [27]. The majority of ATP utilized by the cardiomyocyte is used for myosin ATPase of the contractile filaments, the sarcolemmal Na^+^/K^+^-ATPase and the Ca^2+^-ATPase of the sarcoplasmic reticulum (SR) [28].

Cardiomyocytes rely on the aerobic metabolism, and the vast majority of ATP is synthesized in the mitochondria. The initial substrates for ATP synthesis include glucose and fatty acids. The mitochondrial respiration and beta-oxidation of fatty acids occur in the mitochondria and require constant delivery of oxygen [29,30]. The cytoplasmic phase, glycolysis, is anaerobic and it provides only a small amount of ATP. Its product, the pyruvate, is transported to the mitochondria and decarboxylated to form acetyl-coenzyme A (acetyl-CoA). Acetyl-CoA is also the end product of mitochondrial fatty-acid beta-oxidation. Next, acetyl-CoA acts as a donor of acetyl groups for the tricarboxylic acid cycle (TCA). Oxidation of the substrates provides energy and reduced nicotinamide adenine dinucleotide (NADH) and flavin adenine dinucleotide (FADH_2_). These two compounds serve as electron donors for the mitochondrial electron transport chain (ETC). The flow of electrons along the ETC components, which have a gradually increasing redox potential, provides energy for the transfer of protons across the inner mitochondrial membrane. Protons collected in the intermembrane space generate the electrochemical gradient that is called the inner mitochondrial membrane (IMM) potential (ΔΨm). ATP synthase located in the cristae of IMM uses the energy stored in this gradient to drive ATP synthesis in the process of oxidative phosphorylation [31].

In addition to involvement in ATP synthesis, mitochondria are an important regulator of intracellular calcium balance. The increased concentration of calcium ions in the cytoplasm increases its electrochemical gradient between the cytoplasm and the matrix, and promotes the influx of calcium via the mitochondrial calcium uniporter located in the inner mitochondrial membrane into the mitochondria. In this way, mitochondria can buffer the excess of cytosolic Ca^2+^ or release it via the sodium/calcium symporter (NCLX) when its concentration in the cytoplasm falls down [32].

## 4. Involvement of Mitochondrial Dysfunction in the Pathogenesis of AF

Recent investigations link mitochondrial dysfunction with the pathogenesis of AF [33]. Frequent depolarizations of atrial myocardium increase energetic demands of the cells. In paroxysmal or short-lasting persistent AF, mitochondria are able to increase the synthesis of ATP, but with time the production of ATP decreases, suggesting mitochondrial dysfunction [33]. Low ATP levels affect the intracellular ionic equilibrium, decrease the efficiency of all energy-requiring enzymatic reactions and impair contraction, relaxation and ionic homeostasis of the cell. Diminished levels of ATP lead to activation of cytoplasmic glycolytic enzymes and increased synthesis of lactate that may be considered a mechanism similar to the Warburg effect found in rapidly growing tumors [34,35]. Cellular metabolic stress results in the lowering of the ATP/AMP ratio, which activates the energy sensor adenosine monophosphate protein kinase (AMPK). Activation of this enzyme shifts metabolic pathways toward glycolysis and inhibits the anabolic processes. AMPK can also affect the ionic channels, for example, the ATP-sensitive potassium channel and slow inward calcium channel, and thus modify electrophysiological properties of the cardiac myocytes [14,36]. AMPK is activated in the atrial myocardium in paroxysmal but not long-lasting AF, and is considered the compensatory response to the metabolic stress induced by the arrhythmia [14].

Dysfunctional mitochondria are the source of a large amount of free radicals, especially the superoxide anion (O_2_^−^), which oxidizes many intracellular targets including RyR2 of the SR and the sarcolemmal inward sodium channel [37]. These changes directly alter cardiomyocyte’s excitability and intercellular coupling and build-up the functional background to maintain the reentrant circuits. In addition, mitochondrial dysfunction leads to cytokine release, activation of fibroblasts, deposition of the connective tissue and enhancement of automaticity, promoting development of the arrhythmia [38]. Indeed, experimental and clinical data reveal the association between altered mitochondrial function and the risk of atrial fibrillation [39].

### 4.1. Mitochondrial Ultrastructural Abnormalities

Mitochondrial dysfunction is characterized by both functional and morphological changes. Modification of mitochondrial shape, volume and remodeling of the cristae ultrastructure is coupled to cellular bioenergetic balance and requires the involvement of several mitochondrial proteins [40]. A rapid depolarization rate during AF increases demands for the generation of high-energy compounds and implies additional stress to the cardiomyocytes.

Persistent atrial fibrillation induced in goat initially induced degradation of myofibrils and accumulation of glycogen that were followed by mitochondrial elongation and changes in the cristae orientation; however, no further ultrastructural mitochondrial changes were evident after 16 weeks of the arrhythmia in this animal model [41]. In the mouse with AF and HF, atrial cardiomyocytes contained defective mitochondria with matrix edema and interruption of the inner and outer membrane structures that were related to diminished ATP synthesis [42]. Ozcan et al. described increased volume and number of mitochondria in AF accompanying HF in mice.

In atrial samples collected from patients during cardiac surgery, the ultrastructural examination suggested an increased number of mitochondria frequently having changed shape during AF, but without obvious swelling and cristae derangement [43]. In the model of atrial tachycardia using human atrial samples subjected to rapid pacing, there was an increased number of swollen mitochondria with partial cristaeolysis and completely disrupted mitochondria, which were prevented by verapamil, which suggests calcium-related mechanisms of these ultrastructural changes [44]. Indeed, elevated sarcoplasmic Ca^2+^ levels, secondary to Ca^2+^ leak from the SR in RyR2 mutant mice, leads to mitochondrial dysmorphology [45]. The mitochondrial matrix volume can be additionally influenced by potassium fluxes through the Ca^2+^-dependent (K-Ca) and ATP-dependent potassium channels (mitoK_ATP_) that promote edema, whereas K^+^/H^+^ exchanger (KHE) allowing for potassium efflux prevents mitochondrial swelling [46].

### 4.2. Disturbed Mitochondrial Biogenesis

Mitochondrial biogenesis is a complex process of increasing the global mass of mitochondria within the cell. The process of mitochondrial biogenesis includes the fusion and fission events and concomitant control of mitochondria quality.

The primary mitochondrial biogenesis is induced by environmental stress, such as exercise, caloric restriction, low temperature, oxidative stress, cell division, renewal and differentiation, through the cyclic adenosine monophosphate (cAMP) and protein kinase A (PKA) pathways.

The biogenesis of mitochondria is strictly connected with the mitochondrial ultrastructural changes and requires involvement of many regulatory proteins, which are involved in the division (fission) and contribute to a build-up of the structural and functional basis. The majority of proteins are encoded by nuclear DNA, but some of them are based on autonomous mitochondrial DNA [47,48]. The master regulator of mitochondrial biogenesis is PGC-1α (peroxisome proliferator-activated receptor-γ coactivator 1-α, the product of the PPARGC1A gene). High energetic demand augments the expression of PGC-1α [49]. Activation of PGC-1α is achieved through its deacetylation by sirtuin 1 (SIRT1) or phosphorylation by AMPK and is inhibited by histone acetyltransferase GCN5 (general control of amino-acid synthesis 5) [47,49]. PGC-1α directly adjusts transcription, ribosome formation and assembly of the mitochondrial structural proteins, but also stimulates expression of the nuclear respiratory factors 1 and 2 (NRF-1 and NRF-2), through which it promotes the mitochondrial biogenesis indirectly [50]. PGC1-α together with NRFs promote transcription of mitochondrial genes, starting with the activation of mitochondrial transcription factors, such as mitochondrial transcription factor A (Tfam), Yin-Yang 1 (YY1), mitochondrial DNA-directed RNA polymerase (POLRMT), mitochondrial dimethyladenosine transferase 1 (Tfb1m) and transducer of regulated cAMP response element-binding protein (TORC) [51].

In a rabbit model of pacing-induced AF, mitochondrial DNA content and the expression of transcription factors involved in mitochondrial biogenesis were decreased [52]. In another study, examining rats with streptozotocin-induced diabetes mellitus, Shao et al. found impaired expression of the regulators of mitochondrial biogenesis in diabetic animals that was related to high inducibility of atrial fibrillation, and pharmacological improvement of the indices of mitochondrial biogenesis blunts propensity to AF [39]. Impaired expression of PGC-1α was also found among other disturbances before development of postoperative AF [53].

### 4.3. Mitochondria-Related Oxidative Stress

Reactive oxygen species (ROS) in normal cardiac myocytes originate from mitochondria [54]. The electron transfer chain is the source of small amounts of ROS even under physiological conditions, but any disturbance in the proton gradient across the inner mitochondrial membrane leads to diminished ATP and excessive ROS generation [42].

Atrial fibrillation is characterized by excessive ROS generation due to mitochondrial dysfunction but also due to activation of other mechanisms, such as NADPH oxidase [45,55,56,57,58]. In the atrial muscle of patients with AF, reduction of complex I and II activity with increased activity of complex V was found, which was paralleled by increased production of superoxide [59]. Excessive ROS production and diminished superoxide dismutase promoting oxidative stress in atrial samples were also found in patients in whom AF developed during postoperative follow-up [44,55]. Oxidative stress was linked to changes in gene transcription, damage to mitochondrial DNA, increased activity of NADPH oxidase and xanthine oxidase and local induction of inflammatory processes. Oxidation of ryanodine receptors causes their dysfunction and leakage of Ca^2+^ from the SR [60]. On the other hand, disturbed intracellular Ca^2+^ homeostasis, occurring due to genetic manipulations or under metabolic stress, is related to increased synthesis of ROS by mitochondria [45]. Oxidative stress induces proinflammatory pathways via activation of NF-κB, caspase-1 and NLRP-3 inflammasome [44,61,62]. Moreover, both oxidative stress and inflammation upregulate expression of transforming growth factor β1 (TGF-β1) that leads to proliferation of fibroblasts, their recruitment into myofibroblasts and fibrosis of the atrial myocardium, which is an important constituent of atrial structural remodeling promoting AF [30].

AF development and atrial remodeling can be successfully prevented by treatment with an antioxidant probucol, which in addition to attenuated oxidative stress, also inhibits NF-κB and proinflammatory and profibrotic cytokines release [63]. Potentiation of mitochondrial free radical scavenging mechanisms induced by overexpression of catalase in transgenic mice was sufficient to prevent mitochondrial dysmorphology, SR Ca^2+^ leak and inducibility of AF [45]. Attenuation of oxidative stress by the silencing of the NADPH oxidase 2 with a gene-based approach successfully prevented the electrical remodeling of atrial myocardium and maintenance of the arrhythmia in a canine model of AF [64], and gene therapy directed at the improvement of mitochondrial antioxidative capacity may also be effective in prevention of AF.

## 5. Pharmacological Interventions Improving Mitochondrial Function in AF

**Figure 1:**
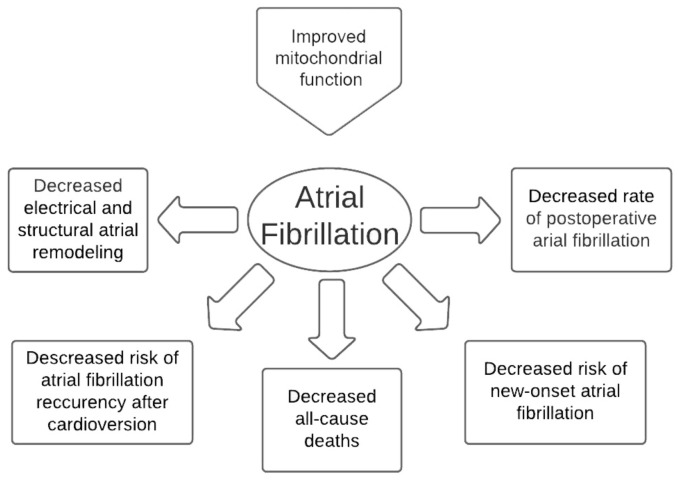
Clinical benefits expected from improvement of the mitochondrial function in atrial fibrillation.

Among pharmacological agents that improve the function of mitochondria and the energetic balance of cardiomyocytes, there are several groups of drugs used in the contemporary practice, mostly in the treatment of diabetes mellitus, and newly developed medications targeting mitochondria more specifically. The clinical benefits achieved by improvement of the mitochondrial function are presented in Figure 1. This chapter describes the effects of these drugs on the mechanisms related to atrial fibrillation.

### 5.1. The Dipeptidyl Peptidase-4 (DDP-4) Inhibitors

The Dipeptidyl peptidase-4 (DDP-4) inhibitors are a new group of oral antidiabetic drugs acting on the incretin system. DDP-4 is an enzyme that inactivates gastric inhibitory polypeptide (GIP) and glucagon-like peptide-1 (GLP-1). Blocking DDP-4 increases the levels of the above hormones, inhibits glucagon and upregulates insulin release.

In cardiomyocytes, hypoxia increases the expression of DDP-4, which triggers the generation of free radicals and ΔΨm reduction [65]. Thus, the cardioprotective effects of DDP-4 are related to attenuation of oxidative stress and amelioration of mitochondrial function [65]. A DDP-4 inhibitor, alogliptin, was shown to reduce the duration of burst pacing-induced atrial fibrillation by 73% in the rabbit model of HF [66]. The exact cellular mechanisms were not evaluated, but alogliptin reduced the extent of atrial fibrosis, improved left atrial capillary density and promoted the activity of endothelial NOS [66]. In another animal study using the model of alloxan-induced diabetes mellitus, treatment with alogliptin resulted in improved ΔΨm and mitochondrial biogenesis via activation of the PGC-1α/NRF1/Tfam signaling pathway that was paralleled by preservation of the left atrial diameter, lowering of hs-CRP levels, upregulation of superoxide dismutase and improved atrial electrical function [67]. Linagliptin, another DDP-4 inhibitor, prevented atrial electrical remodeling, reduced oxidative stress and suppressed AF inducibility in a canine model of atrial fibrillation [68]. The protective effects of DDP4 inhibitors against AF seems to be related to lowering ROS, preserving mitochondrial biogenesis and reducing inflammation [69]. The therapeutic potential of DDP4 inhibitors was confirmed in a large observational study involving over 90,000 diabetic patients, in whom the addition of a DDP-4 inhibitor as a second-line anti-diabetic treatment reduced new onset AF by 35% [70].

### 5.2. Selective Inhibitors of the Sodium-Glucose Co-Transporter 2

Gliflozines are selective inhibitors of the sodium-glucose co-transporter 2 (SGLT2) initially introduced for the treatment of type 2 diabetes mellitus. However, recent studies revealed the cardioprotective potential of these medications. The EMPA-REG OUTCOME trial showed that among patients with DM and HF, SGLT2 inhibitors lead to a significant reduction of hospitalization rate by 35% and cardiovascular deaths by 38% [71]. The CVD-REAL study compared the influence of newly initiated antidiabetic therapy with SGLT2 inhibitors versus other glucose-lowering drugs in a huge cohort of patients with diabetes. The SGLT2 inhibitor was superior to other antidiabetic treatments, decreasing the risk of HF-related hospitalization rates by 39%, all-cause mortality by 51% and combined HF hospitalizations and all-cause death by 46% [72].

The beneficial cardiovascular outcomes initiated further research aimed at establishing the mechanisms of the cardioprotective action of SGLT2 inhibitors. Kidney-dependent volume contraction resulting from glycosuria and osmotic diuresis is the obvious candidate for cardioprotection, but there may also be vascular and direct cardiac actions of these medications that significantly contribute to the observed advantages.

SGLT2-i can reduce arterial resistance, which is related to flow-mediated dilation, suggesting improvement of endothelial function [73]. Research performed by Zhou et al. indeed showed the ameliorating effect of the SGLT2 inhibitor on endothelial cells. In mice with streptozotocin-induced diabetes, treatment with empagliflozin preserved cardiac microvascular barrier function and integrity, sustained eNOS phosphorylation and endothelium-dependent relaxation, as well as higher microvessel density and perfusion. Inhibition of mitochondrial fission, induced by activation of AMPK and attenuated ROS production, were proposed as the mechanism for the observed endothelial effects [74]. 

Direct effects of SGLT2-i on cardiomyocytes were described in both animal and human studies. SGLT2 inhibitors block an Na^+^/H^+^ exchanger (NHE-1) and normalize cytosolic Na^+^ and Ca^2+^ concentrations [75] independently from the presence of DM. Elevated cytoplasmic sodium levels may negatively affect energy supply and demand matching and can even induce mitochondrial oxidative stress [76]. Low cytosolic sodium augments mitochondrial Na^+^/Ca^2+^ exchange, increases mitochondrial calcium concentration and improves mitochondrial function [77]. Prompt binding and blocking of the NHE-1 by SGLT2 inhibitors were proposed, but pharmacological approaches also suggest an indirect effect on the exchanger via the stimulation of AMPK [78].

The results of improved mitochondrial function after empagliflozin treatment were shown in the rat model of STZ-induced diabetes [39]. Shao et al. observed in their study that SGLT2 inhibition restores the mitochondrial ΔΨm and mitochondrial respiratory rate with concomitant increased expression of PGC-1α, NRF-1 and Mfn-1, suggesting promotion of mitochondrial biogenesis. Ameliorated mitochondrial function was related to attenuation of the synthesis of ROS, lower systemic inflammation, inhibition of atrial fibrosis and cardiomyocyte hypertrophy [39]. Involvement of these mechanisms resulted in the reduction of tachypacing-induced AF susceptibility by about 50% in the group treated with empagliflozin [39]. In another experimental study with empagliflozin, Li et al. showed its suppressing activity on oxidative stress and myocardial fibrosis through inhibition of the TGF-β/Smad pathway and activation of Nrf2/ARE signaling [79].

Clinical data from patients with diabetes and present AF show benefits of treatment with SGLT2 inhibitors. The EMPA-REG OUTCOME trial subanalysis revealed that the use of the SGLT2 inhibitors in these patients brings 50% reduction of all-cause deaths, 48% reduction of cardiovascular deaths and significantly decreases the risk of new edema or nephropathy development [80]. A recent analysis of pooled nine cardiovascular and renal outcome clinical trials conducted in patients with and without diabetes revealed 21% relative risk reduction of AF in patients treated with SGLT2 inhibitors as compared to placebo [81].

SGLT2 inhibitors seem to be more effective in reducing the risk of AF than DDP-4 inhibitors in patients with diabetes. The observational study showed that treatment with SGLT2-i was associated with a 39% lower risk of new-onset AF as compared to treatment with DPP4 inhibitors [82].

To date, the majority of both experimental and clinical studies with gliflozins were conducted in diabetic subjects. The improvement of mitochondrial function and cellular energetics, protecting against the development of electrical and structural atrial remodeling, brought by SGLT2 inhibitors is clear in diabetics. The recent study showed that the cardioprotective effect of gliflozins is independent from glycated hemoglobin A1c concentration or from pre-existence of other comorbidities, such as AF, HF or atherosclerotic cardiovascular disease [83]. However, the exact mechanism in which SGLT2 inhibitors reduce the risk of AF in patients without diabetes was not established and requires further investigation [81].

### 5.3. Ubiquinone

Ubiquinone (coenzyme Q10, CoQ10) is an important mitochondrial cofactor involved in the electron transport from complex I to complex II and from complex II to complex III of the respiratory chain. In addition, CoQ10 is an effective antioxidant, membrane stabilizer, cofactor of mitochondrial uncoupling proteins, a stabilizer of calcium-dependent channels, metabolic regulator and an indirect regulator of signaling molecule formation and cell growth [84]. The levels of CoQ10 in the myocardium are relatively high, but can decrease with age, statin treatment or due to genetic defects. Diminished plasma CoQ10 levels are seen in advanced HF and are associated with the severity of symptoms [85]. Although the majority of studies have looked at the effect of CoQ10 treatment in HF, there are premises for considering CoQ10 in AF prevention [86].

Atrial samples collected from patients after a two-week CoQ10 treatment showed better respiratory function and lower levels of oxidative stress marker malonyldialdehyde [87]. In patients with HF, treatment with CoQ10 significantly reduced the incidence of AF during a 12-month follow-up, which was attributed to its antioxidative capacity [88]. In a small, double-blind, randomized controlled trial, short-term CoQ10 treatment reduced incidence of postoperative AF by about half [89]; however, these results were not confirmed by other studies, as summarized by the meta-analysis by de Frutos et al. [90].

Taking into account a good tolerability and favorable safety profile, CoQ10 can be considered an adjuvant therapy reducing the risk of AF in certain situations; however, further studies are required to elucidate its clinical effectiveness.

### 5.4. Metformin

Metformin, the first-line antidiabetic medication, can prevent AF by attenuation of atrial remodeling [91,92]. The analysis of data from 645,710 patients with type 2 DM during a 13-year follow-up collected in the Taiwan National Health Insurance Research Database showed that metformin decreased the incidence of AF by 19% [93]. The mechanisms of this clinical effect are not clear and a possible explanation is brought by experimental studies with pacing-induced AF. In one study, metformin activated AMPK, Src kinase and normalized connexin expression to attenuate the pacing-induced increase in the refractory period, AF inducibility and AF duration [94]. In another study it prevented atrial electrical and structural remodeling by activating the AMPK/PGC-1α/PPAR-α pathway and normalizing the metabolic pathway activities [92]. Due to its electronegativity, metformin becomes concentrated in mitochondria and, thus, orchestration of cellular energy metabolism may play a role in AF development. Preservation of mitochondrial function by metformin was related to improved oxygen consumption and enhanced function of complexes I, II and IV [95]. In addition, in rodents subjected to myocardial infarction, metformin improved cardiac function via preservation of mitochondrial respiration and mitochondrial biogenesis, which was paralleled by upregulation of PGC-1α [91].

Metformin remains the first choice of treatment in DM, and with no doubt, it reduces cardiovascular risks in diabetes patients. Animal experiments suggest that metformin has cardioprotective effects, including prevention of AF, even without DM. The question remains whether metformin has a significant cardioprotective effect also in people without diabetes.

### 5.5. Thiazolidinediones

Thiazolidinediones (TZDs) are the next group of medications used in treatment of DM influencing mitochondrial function, introduced as agonists PPAR-γ to reduce insulin resistance. In an experimental model of alloxan-induced DM in rabbits, TZDs attenuated atrial remodeling and AF inducibility and improved function of ion channels (I_Ca_ and I_Na_) and activation of pERK, TGF-β1, TLR4, NF-κB and HSP70 [96]. In the Wistar AF model, pretreatment with pioglitazone decreased AF duration, and upregulation of antioxidant mechanisms and inhibition of mitochondrial apoptotic signaling pathways were proposed as the protective mechanisms [97]. A few registries and clinical trials suggest the effectiveness of TZD in preventing atrial fibrillation. The analysis of Danish nationwide registries of over 100,000 diabetic patients with no prior AF showed that TZD reduced the incidence of AF by 24% when adjusted for age, sex and comorbidities, compared to other second-line antidiabetic treatments [98]. Another suggestion for TZDs preventive potential against AF was provided by a meta-analysis of three randomized clinical trials and four observational studies, in which TZDs reduced the risk of AF incidence by 23% (for new-onset AF) and provided a 59% risk reduction of AF recurrence [99]. Nevertheless, in patients with coronary artery disease treated with either TZD or other second-line medications during a median follow-up of 4.2 years, TZDs did not affect the prevalence of AF [100]. Furthermore, TZDs improved the recurrence of AF after electrical cardioversion in a small randomized prospective study [101]. Thus, there is a need for prospective randomized trials to determine the usefulness and safety of TZDs in AF prevention.

### 5.6. Fibrates

Fibrates are agonists of PPARα, and they are used in treatment of hypertriglyceridemia via attenuation of hepatic apoC-III and promotion of lipoprotein lipase-mediated lipolysis [102]. They may also influence mitochondrial function through the PPARα/PGC-1α pathway [103]. In the experimental animal model of AF, fenofibrate was found to decrease metabolic remodeling by regulating the PPAR-α/sirtuin 1/PGC-1α pathway and reversed shortened atrial refractory period [104]. Bezafibrate induced beneficial effects on mitochondrial biogenesis, increasing expression of PPARGC1A, GFAP, S100B, DCX NRF1 and TFAM genes and an mtDNA copy number [105]. The lipid lowering medications, including fibrates, were connected with a significant decrease in AF prevalence in patients with reduced left ventricular ejection fraction, independently from lipid profile, possibly via anti-inflammatory and antioxidant effects [106]; however, the clinical benefits of this treatment for AF outcomes were not evaluated.

### 5.7. Trimetazidine

Trimetazidine (TMZ) is an anti-anginal drug that was approved for the treatment of ischemic heart disease, and its beneficial effects are related to improvement of cellular energetic balance. TMZ inhibits the long-chain 3-ketoacyl CoA thiolase, which is involved in fatty acid oxidation. It was originally proposed that inhibition of beta-oxidation shifts mitochondrial substrate utilization toward glucose and improves ATP synthesis; however, recent studies challenge this traditional paradigm, showing lack of changes in fatty acid or carbohydrate metabolism in the myocardium in either acute or chronic treatment with TMZ and suggest other mechanisms to be responsible for the protective activity of this drug [107,108].

In ischemic conditions, TMZ directly acts on the respiratory chain activity via activation of the complex I [109,110]. Improved function of the ETC increases consumed the ADP/O_2_ ratio and reduced the generation of ROS [111]. In ischemic conditions, TMZ normalizes the expression of factors regulating mitochondrial biogenesis, such as PPARγ and PGC-1α, and also adjusts the expression of Mfn-1, Drp1 and Opa-1 to the normal levels, which suggests the impact on the mitochondrial fusion/fission dynamics [110]. TMZ was, however, not proven to induce any beneficial effects on the mitochondrial function without the ischemic context, but regardless of the precise metabolic mechanisms of action, its antiarrhythmic activity was postulated. In a series of studies, TMZ improved the electrocardiographic parameters reflecting susceptibility to atrial and ventricular arrhythmias, such as P-wave duration and P-wave dispersion [112], heart rate variability [113] and Tpeak–Tend duration and dispersion [114]. The preliminary report using the tachypacing model suggests that TMZ prevents tachycardia-induced atrial ultrastructural remodeling, decreases AF inducibility and shortens AF duration [112,115]. It is unknown if these protective effects of TMZ in conditions without an ischemic context are related to improvement in mitochondrial function. In a chronic atrial pacing model in dogs, TMZ did not affect the levels of creatine phosphate, ATP, ADP, AMP and total adenosine, and it also did not prevent ultrastructural changes, such as mitochondrial swelling and cristae derangements, myolysis and karyopynkosis, but reduced oxidative stress and promoted endothelial NO synthase expression [116,117].

The exact mechanisms of arrhythmic substrate modulation and clinical usefulness of TMZ in AF remain uncertain and require further studies.

### 5.8. Ranolazine

Ranolazine is an anti-anginal drug, which also has anti-arrhythmic properties due to selective inhibition of sodium currents [118]. As shown in the metanalyses, ranolazine decreases the probability of AF development in different clinical settings by about 50%, increases the cardioversion success rate of amiodarone and decreases time to restoration of the sinus rhythm [119,120]. The proposed mechanism of action of ranolazine is the prolongation of post-repolarization refractoriness and the slowing of the conduction velocity [121]. In addition to these electrophysiological effects, it was shown that ranolazine improves mitochondrial function, attenuates oxidative stress, suppresses apoptosis through augmentation of the Bcl-2/Bax ratio, reduction of the cleaved caspase-3 level and activation of the Akt/mTOR signaling pathway [122].

### 5.9. Experimental Treatments Targeting Mitochondria

Elamipretide (other names include Bendavia, MTP-131 or SS-31) is the first in the class of mitochondria-targeted drugs that entered clinical trials for treatment of HF. Elamipretide improves mitochondrial energetics and decreases generation of reactive oxygen species, possibly by stabilizing the mitochondrial membrane and cytochrome c [123]. Cardioprotective effects of the drug were shown in basic and human studies. In a dog model of chronic heart failure of ischemic etiology, elamipretide restored mitochondrial state-3 respiration, ΔΨm, rate of ATP synthesis, normalized the ATP/ADP ratio, reduced levels of TNF-α and C-reactive protein and prevented decline of left ventricular systolic function [124]. Elamipretide treatment of samples of failing human ventricular myocardium significantly improved mitochondrial oxygen flux, complex I and complex IV of the ETC activities, and ETC-supercomplex-associated complex IV activity [123]. An initial randomized clinical trial using single elamipretide infusion in patients with heart failure with reduced ejection fraction demonstrated significant reduction of left ventricular end-diastolic volume following infusion [125]. Despite encouraging initial results, a recent phase 2 clinical trial on a small cohort of patients with heart failure showed a lack of improvement of left ventricular ejection fraction after repeated elamipretide administration for 28 days [126].

A few more medications specifically targeting mitochondria are currently being examined regarding their safety and efficacy. The potential of these still experimental therapies supporting mitochondrial function in the prevention of AF should be investigated after finishing outgoing safety and efficiency measurements. The list of these medications with a short description is provided in Table 1.

KL1333 increases NAD^+^ levels and activates SIRT1/AMPK/PGC-1α signaling, improves mitochondrial function and decreases oxidative stress in fibroblasts from mitochondrial encephalomyopathy, lactic acidosis and stroke-like episodes in patients [129].

Another medication, KH176, can effectively reduce increased cellular ROS levels and protect oxidative phosphorylation deficient primary cells against redox perturbations by interacting with the thioredoxin system/peroxiredoxin enzyme machinery [130].

**Figure 2:**
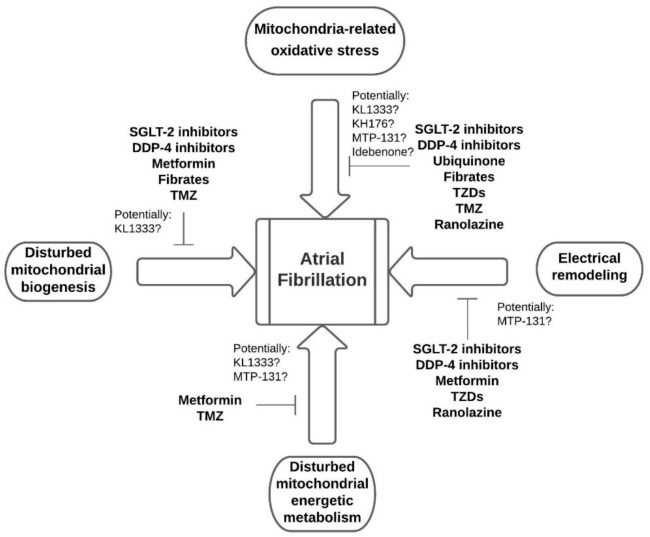
Pharmacological interventions targeting basic mechanisms of atrial fibrillation. Abbreviations: SGLT2—sodium-glucose co-transporter 2, DDP-4—dipeptidyl peptidase-4, TZDs—thiazolidinediones, TMZ—trimetazidine.

Idebenone is a synthetic coenzyme Q10 analog and was tested to treat diverse diseases in which mitochondrial function is impaired [131]. It showed some cardioprotective potential in the animal model of ischemia/reperfusion, but other effects in heart diseases need to be examined [132]. The summary of the medications acting on the basic mechanisms linking mitochondrial dysfunction with atrial fibrillation is presented in Figure 2.

The impact of these medications targeting mitochondria in AF risk reduction or improvement of the AF outcomes are unknown. Most of the few trials conducted to date excluded patients with AF; thus, further basic and clinical investigations are required to establish the usefulness of these medications in patients with AF.

Gene therapy is another potential therapeutic approach; however, to date, it remains in an experimental phase. Genetic constructs, mostly inserted in adenoviral vectors, may be delivered to the myocardium by direct intramyocardial injection, by epicardial gene painting or via intracoronary infusion [133]. These gene-based approaches in animal models of AF were successful in the restoration of the sinus rhythm or improved control of the ventricular rate. Nevertheless, it is still too early for their introduction into clinical practice [133,134,135].

## 6. Conclusions

Preserved mitochondrial function is crucial for cardiomyocyte integrity and uncompromised cardiac performance. Atrial fibrillation may result from, induce or exaggerate energetic imbalance, disturbed metabolism and oxidative stress, and all of them are related to mitochondrial dysfunction, which can be targeted by currently available medications such as metformin, thiazolidinediones, fibrates, ranolazine or selective inhibitors of the sodium-glucose co-transporter 2. However, there is a gap in the knowledge about the significance of the effects in atrial fibrillation prevention and the impact of these treatments on the outcomes of the arrhythmia. Randomized double-blind placebo-controlled trials are needed for medications affecting the mitochondrial function in order to allow for more wide indications in clinical practice. The possible indications could include preservation of sinus rhythm in individuals with paroxysmal AF, or, after cardioversion, prevention or treatment of post-operative atrial fibrillation (POAF) with usage as a longitudinal additive in patients with persistent or permanent AF.

## Figures and Tables

**Table 1:** Experimental medications targeting mitochondria. The efficacy of these medications in the treatment of AF was not tested. The clinical trial identifiers in the Clinicaltrial.org database are provided in the “References” column.

Medication	Mechanism of Action	Target Diseases	Current Research Stage	References
Elamipretide (MTP-131)	Improves mitochondrial ultrastructure and bioenergetics	Primary Mitochondrial Myopathy	Experimental studies; phase 3 randomized, double-blind, placebo-controlled trial—terminated; phase 2 randimized trials in heart failure—completed	Clinical trial, identifier: NCT03323749 NCT02788747 NCT02814097 NCT02914665
KL1333	The safety and efficiency measurements in progress, with potential effects on NAD+/NADH, FGF21 and GDF15 concentrations	Primary mitochondrial disease	Phase Ia/Ib trial (recruiting)	Clinical trial, identifier: NCT03888716
KH176	ROS level reduction and cell protection against redox stress	Mitochondrial disease	Phase IIb open-label, multi-center trial (planned ending date: June 2021)	Clinical trial [107,108] identifier: NCT02544217; NCT02909400; NCT04165239 NCT04604548
REN001	Selective PPAR delta agonist	Primary Mitochondrial Myopathy	Finished phase I due to COVID-19 pandemic but with sufficient data gathered to achieve the study objective	Clinical trial, identifier: NCT03862846
Idebenone	Antioxidant with ATP preserving properties: stimulates mitochondrial electron flux, increases respiratory function	Friedreich ataxia (FRDA) and Duchenne muscular dystrophy	Finished trial phase III in Duchenne muscular dystrophy and phase III study (IONIA)	[127,128]

## Data Availability

Not applicable.
